# Deep brain stimulation improves central nervous system inflammation in Parkinson's disease: Evidence and perspectives

**DOI:** 10.1111/cns.14167

**Published:** 2023-03-21

**Authors:** Lei Chang, Wen‐Wen Dong, Bei Luo, Chang Qiu, Yue Lu, Xing‐Jian Lin, Wen‐Bin Zhang

**Affiliations:** ^1^ Department of Functional Neurosurgery The Affiliated Brain Hospital of Nanjing Medical University Nanjing China; ^2^ Department of Neurology The Affiliated Brain Hospital of Nanjing Medical University Nanjing China

**Keywords:** deep brain stimulation, immunological disturbances, inflammation, Parkinson's disease, RANTES, TNF‐α

## Abstract

**Background:**

In Parkinson's disease (PD), inflammation may lead to the degeneration of dopaminergic (DAergic) neurons. Previous studies showed that inflammatory mediators mainly contributed to this phenomenon. On the other hand, invasive neuromodulation methods such as deep brain stimulation (DBS) have better therapeutic effects for PD. One possibility is that DBS improves PD by influencing inflammation. Therefore, we further explored the mechanisms underlying inflammatory mediators and DBS in the pathogenesis of PD.

**Methods:**

We measured serum levels of two inflammatory markers, namely RANTES (regulated on activation, normal T cell expressed and secreted) and tumor necrosis factor‐alpha (TNF‐α), using Luminex assays in 109 preoperative DBS PD patients, 49 postoperative DBS PD patients, and 113 age‐ and sex‐matched controls. The plasma protein data of the different groups were then statistically analyzed.

**Results:**

RANTES (*p* < 0.001) and TNF‐α (*p* = 0.005) levels differed significantly between the three groups. A strong and significant correlation between RANTES levels and Hoehn‐Yahr (H‐Y) stage was observed in preoperative PD patients (*r*_s_ = 0.567, *p* < 0.001). Significant correlations between RANTES levels and Unified Parkinson's Disease Rating Scale III (UPDRS III) score (*r*_s1_ = 0.644, *p* = 0.033 and *r*_s2_ = 0.620, *p* = 0.042) were observed in matched patients. No correlation was observed for TNF‐α levels.

**Conclusion:**

The results of this study indicate that PD patients have a persistent inflammatory profile, possibly via recruitment of activated monocytes, macrophages, and T lymphocytes to the central nervous system (CNS). DBS was shown to have a significant therapeutic effect on PD, which may arise by improving the inflammatory environment of the central nervous system.

## Graphical abstract

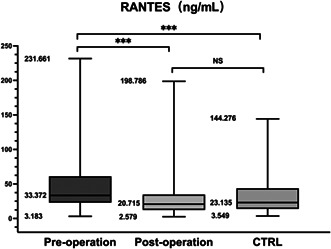

## 1 INTRODUCTION

Parkinson's disease is a common neurodegenerative disorder in middle‐aged and elderly people caused by the death of DAergic neurons in the substantia nigra pars compacta (SNpc), resulting in dopamine deficiency and imbalance of dopamine and acetylcholine neurotransmitter levels. These changes cause motor symptoms such as tremors, myotonia, slow movement, postural instability, and gait difficulty.^1^, ^2^ At present, the specific pathogenesis of PD is unclear. Some scholars believe that autophagy occurs in DAergic neurons in the basal ganglia of PD patients and dysfunction occurs in the functional structure of mitochondria and lysosomes,^3^, ^4^ while others believe that the protein transmitting signals between neurons are also abnormal in PD patients.^5^, ^6^ Recently, neuroinflammation and immunological disturbance theory has become a hotspot in PD research.^7^, ^8^, ^9^, ^10^, ^11^

Early PD is mainly diagnosed by asking patients about their medical history and based on the physician's clinical experience. Most PD patients present after more than 60% of dopaminergic neurons have lost measurable function, leading to a low accuracy of early diagnosis.^12^ Therefore, there is an urgent need to identify reliable markers to diagnose PD and track its progression. Of the current clinical symptomatology, imaging, genomics, and biochemistry markers of PD being explored, biologic markers are potentially the most valuable which are considered to be a breakthrough in studying the pathogenesis of PD.^13^, ^14^, ^15^ With the development of biologic markers of PD, researchers have begun to explore protein levels in blood, urine, saliva, and other body fluids. Blood has become the ideal source for testing PD biomarkers due to its easy collection method, low contamination risk, and low invasiveness. Neuroinflammation in the CNS is characterized by the activation of microglia and astrocytes, which produce various inflammatory factors and chemokines that destroy the blood brain barrier (BBB) and promote the degeneration of dopaminergic neurons.^16^, ^17^, ^18^ The independent or combined action of T cells also participate in this process.^19^ Therefore, pro‐inflammatory factors, such as RANTES and TNF‐α, may be the key to unlocking the pathogenesis of PD. RANTES is a C‐C beta‐chemokine with strong chemoattractant activity for T lymphocytes and monocytes.^20^ Chemokines are released by activated macrophages and microglia in the process of human inflammatory immunity, and inflammatory cells secrete inflammatory factors such as RANTES to recruit additional inflammatory cells to participate in the inflammatory response.^21^, ^22^, ^23^ TNF‐α has potential cytotoxic effects on DAergic neurons and is an important immune signaling molecule. It is mainly produced by astrocytes and microglia in the CNS and can induce the release of neurotoxic substances and the synthesis of inflammatory factors, whose persistent release can aggravate damage to DAergic neurons.^24^

Several studies have reported a link between inflammation and PD. In autopsy studies of PD patients, pro‐inflammatory cytokines, and chemokines, especially RANTES and TNF‐α, showed upregulated expression in brain tissue and cerebrospinal fluid.^25^, ^26^ Tang et al.^27^ showed that inflammatory mediators may reflect the role of systemic inflammation in the neurodegenerative process of PD. The serum RANTES level of PD patients were higher than that of controls, and the RANTES level was strong correlated with H‐Y stage and disease duration in patients. Previous studies have reported significantly higher serum levels of TNF‐α in PD patients compared with controls, but no significant correlation with factors such as severity of disease.^26^, ^28^

Until recently, PD was mainly treated by drugs. However, with the advancement of technology and change in treatment philosophy, novel treatment modalities such as surgery and rehabilitation exercises have emerged. The advantages of DBS over nucleus disruption surgery are that it is highly effective, minimally invasive, reversible, and modifiable. Although DBS can control motor symptoms in PD patients, the complete underlying mechanism of action remains unclear due to the complexity of the brain region environment.

The current study aims to examine changes both in plasma RANTES and TNF‐α levels in PD patients and controls. The study goals are to validate and explore the effects of DBS on plasma biomarkers and examine whether DBS efficacy is related to neuroinflammatory responses and immune imbalance.

## 2 MATERIALS AND METHODS

### 2.1 Patients and controls

For this retrospective study, we recruited 158 PD patients treated at The Affiliated Brain Hospital of Nanjing Medical University from October 2017 to October 2019. All patients were diagnosed with PD according to the Movement Disorder Society Clinical Diagnostic Criteria for PD by two neurologists and met the following criteria: (i) no evidence of active infections or systemic inflammation on any serological examination or clinical examination; (ii) no use of steroid, non‐steroidal, or anti‐inflammatory drugs in the 3 months prior to being recruited; (iii) no evidence of cognitive dysfunction such as dementia, delirium, and mental disorders including anxiety and depressive disorder; and (iv) willing and able to participate in long‐term follow‐up. Depending on the intervention, PD patients were divided into two groups pre‐operation (*n* = 109) and post‐operation (*n* = 49). The control group (*n* = 113) was recruited from the Physical Examination Center of The Affiliated Brain Hospital of Nanjing Medical University. All people in the control group were healthy and had no symptoms of neurodegenerative diseases. This study was approved by the Ethics Committee of The Affiliated Brain Hospital of Nanjing Medical University and all participants provided written informed consent before enrollment.

### 2.2 Sample collection and measurement

Peripheral venous blood samples (5 mL) were collected between 8:00 AM and 10:00 AM from participants in a resting state, following a 12 h washout period (OFF‐medication/ON‐DBS). Blood samples were also collected 1 month after surgery to avoid any influence of postoperative inflammation. The samples were allowed to clot for 30 min at room temperature before centrifugation for 15 min at 3000 rpm. Next, the supernatant was collected, transferred to a 1.5 mL cryopreservation tube, and stored at −80 °C until further analysis. Analysis was performed using Luminex kits obtained from Millipore (Billerica, MA). Assays were performed as per the manufacturer's instructions to determine the plasma levels of 40 proteins. Properly diluted plasma samples were incubated with antibody‐coupled microspheres and then with the biotinylated detection antibody before the addition of streptavidin‐phycoerythrin. The captured bead complexes were measured with an FLEXMAP 3D system (Luminex Corporation) using the following instrument settings: events/bead, 40; sample size, 50 μL; discriminator gate, 8000–15,000. The raw data (mean fluorescence intensity) were collected and further processed to calculate the protein concentration.

### 2.3 Data preprocessing

**FIGURE 1:**
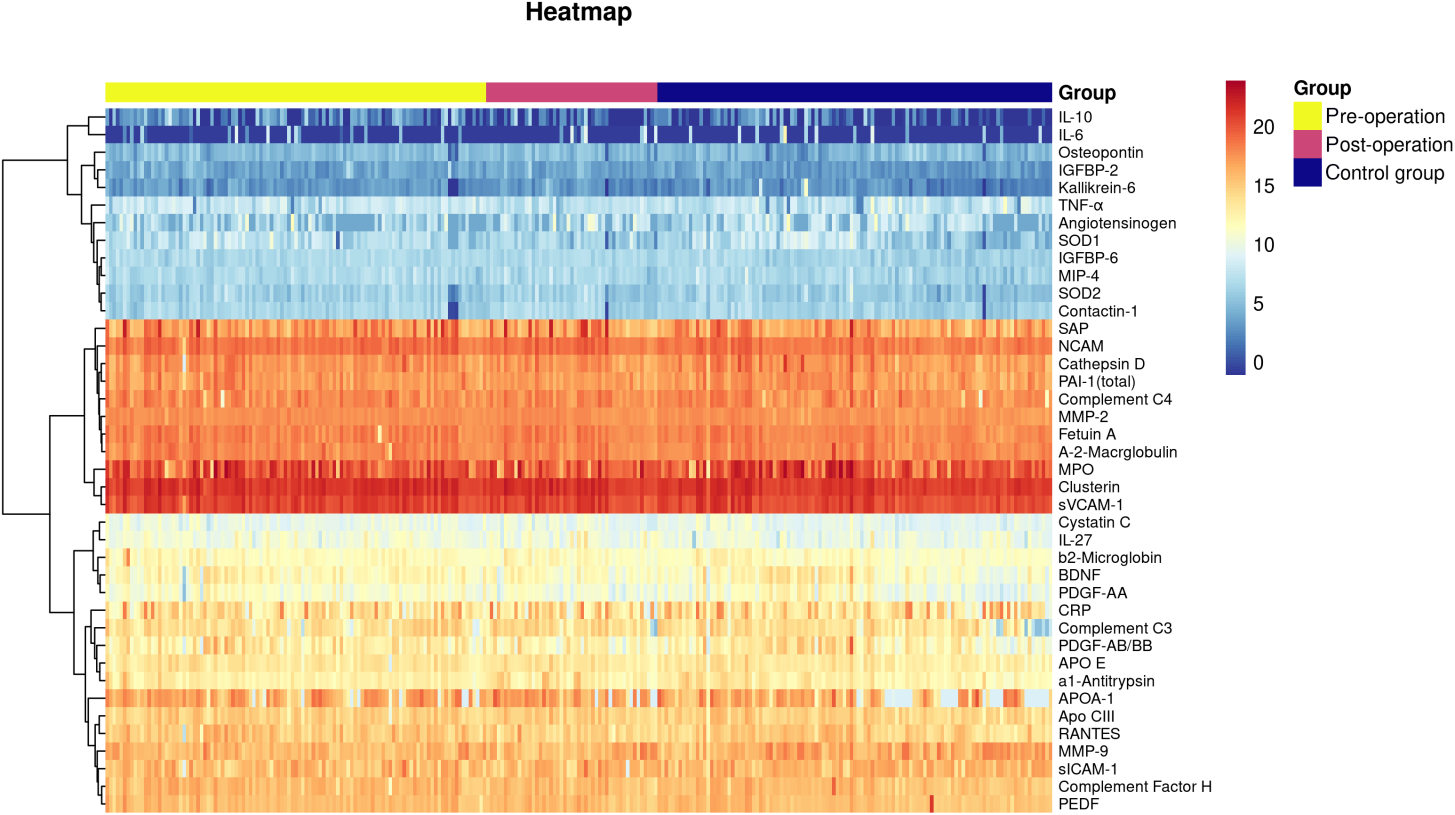
Heatmap shows a differential analysis of 40 plasma proteins using the log_2_ value of the protein content (R software version 4.2.0, package pheatmap version 1.0.12).

**FIGURE 2:**
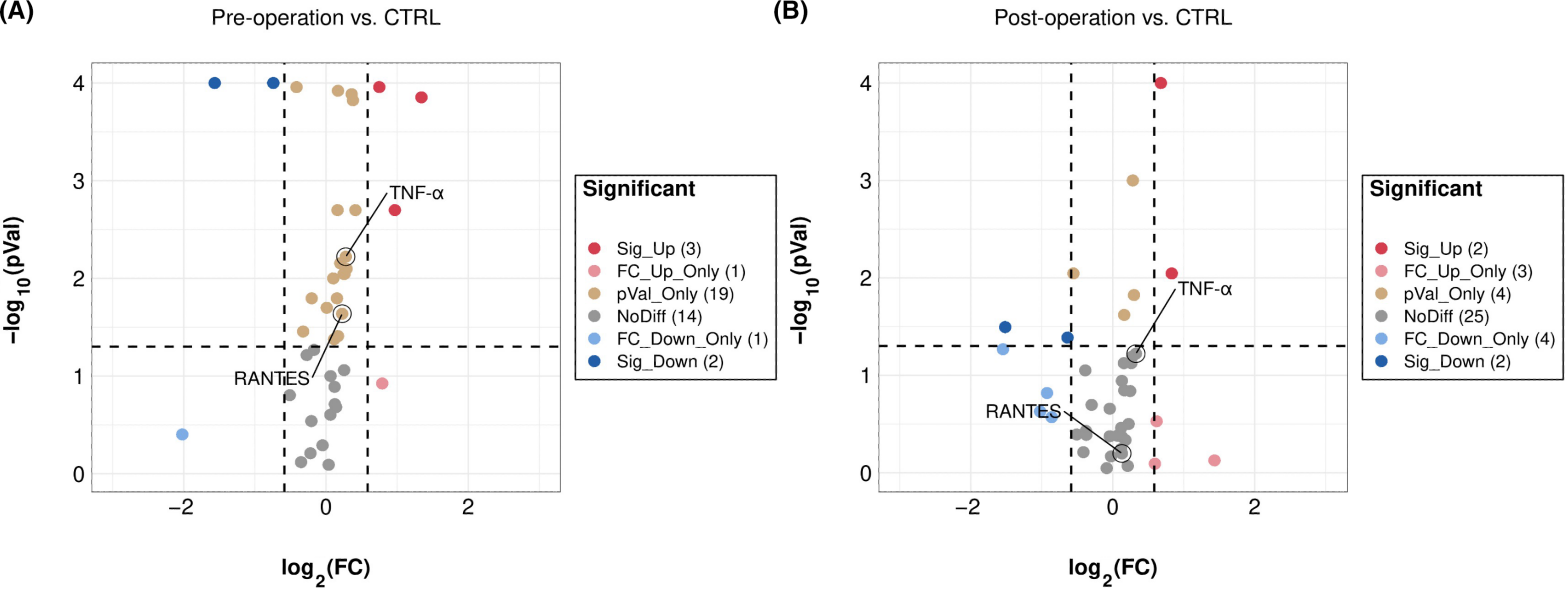
Advanced volcano plot displayed the relationship between PD patients and CTRL. a pre‐operation versus CTRL. It showed that the RANTES and TNF‐α levels were upregulated. b post‐operation versus CTRL. The RANTES and TNF‐α levels were no differential. FC_Down_Only, only fold change down; FC_Up_Only, only fold change up; NoDiff, no differential; pVal_Only, only *p*‐value up; Sig_down, significant down; Sig_Up, significant up.

Before statistical analysis, quality checks (QC) were performed for each assay. The median fluorescent intensity (MFI) was measured using xPONENT 5.1 (Luminex Corporation) and exported into Milliplex Analyst 5.1 (VigeneTech) for estimation of the protein concentrations using a five‐parameter logistic fit. Briefly, all analytes that passed QC checks based on the four criteria (standard curve linearity, intra‐assay coefficient of variation, interassay coefficient of variation for reference sample, and percentage of missing data) underwent further analysis. Next, we performed a differential analysis of 40 plasma proteins using the log_2_ value of the protein content by heatmap (package pheatmap version 1.0.12, Figure 1). The RANTES and TNF‐α levels showed upregulation. The volcano plot displays the relationship between the log_2_ fold change and −log_10_ *p*‐value (package ggplot2 version 3.3.6, Figure 2, Table 1). Differently expressed plasma proteins were screened using a threshold fold change of 1.50. Similar results were observed. Any inconsistencies in the results are explained in the discussion.

**TABLE 1:** Changes in plasma protein levels.

	Pre‐operation vs. CTRL	Post‐operation vs. CTRL
Sig_Up	IL‐6, SAP, BDNF	IL‐6, PDGF‐AB/BB
FC_Up_Only	Fetuin A	Cystatin C, Fetuin A, BDNF
pVal_Only	a1‐Antitrypsin, IL‐27, CRP, MMP‐9, sICAM‐1, Complement C3, Contactin‐1, MMP‐2, PEDF, IGFBP‐2, IGFBP‐6, Cathepsin D, PAI‐1(total), TNF‐α, RANTES, PDGF‐AB/BB, SOD1, Angiotensinogen	a1‐Antitrypsin, CRP, APOA‐1
		SOD2
NoDiff	Kallikrein‐6, NCAM, MIP‐4, SOD2,	SAP, Angiotensinogen, IL‐27, sICAM‐1, Complement C3, Contactin‐1, Kallikrein‐6, NCAM, MIP‐4, Apo CIII, MMP‐2, Clusterin, PEDF, Complement C4, IGFBP‐2, IGFBP‐6, A‐2‐Macrglobulin, APO E, PAI‐1 (total), TNF‐α, RANTES, IL‐10, b2‐Microglobin, SOD1, Cathepsin D
	Apo CIII, Clusterin, Complement C4, A‐2‐Macrglobulin, sVCAM‐1, APO E, MPO, PDGF‐AA, IL‐10, Cystatin C,	
	Complement factor H	
FC_Down_Only	b2‐Microglobin	MMP‐9, sVCAM‐1, MPO, PDGF‐AA
Sig_Down	APOA‐1, Osteopontin	Osteopontin, Complement Factor H

*Note*: Differences in levels of 40 tested plasma proteins.

Abbreviations: FC_Down_Only, only fold change down; FC_Up_Only, only fold change up; NoDiff, no differential; pVal_Only, only *p*‐value up; Sig_down, significant down; Sig_Up, significant up.

### 2.4 Statistical analysis

Shapiro–Wilk's test and Levene's test, respectively, were used to confirm the assumptions of normality and homogeneity of variance for all the variables. Owing to deviations from the normal distribution or heterogeneity of variances, nonparametric tests (Mann–Whitney test, Kruskal–Wallis test) were used to compare RANTES and TNF‐α levels between groups. Student's *t*‐test was used when the data met normality, unless otherwise specified. Spearman's correlation coefficient was used to evaluate the relationships between all data. One‐way analysis of variance (ANOVA) and the chi‐squared test were also used when appropriate. The data are presented as the mean ± standard deviation (SD) when under a normal distribution and the median (interquartile range, IQR) when under a skewed distribution. Results were considered statistically significant at *p* < 0.05. Statistical analyses were performed using SPSS 26 (IBM) and R software (version 4.2.0).

## 3 RESULTS

**FIGURE 3:**
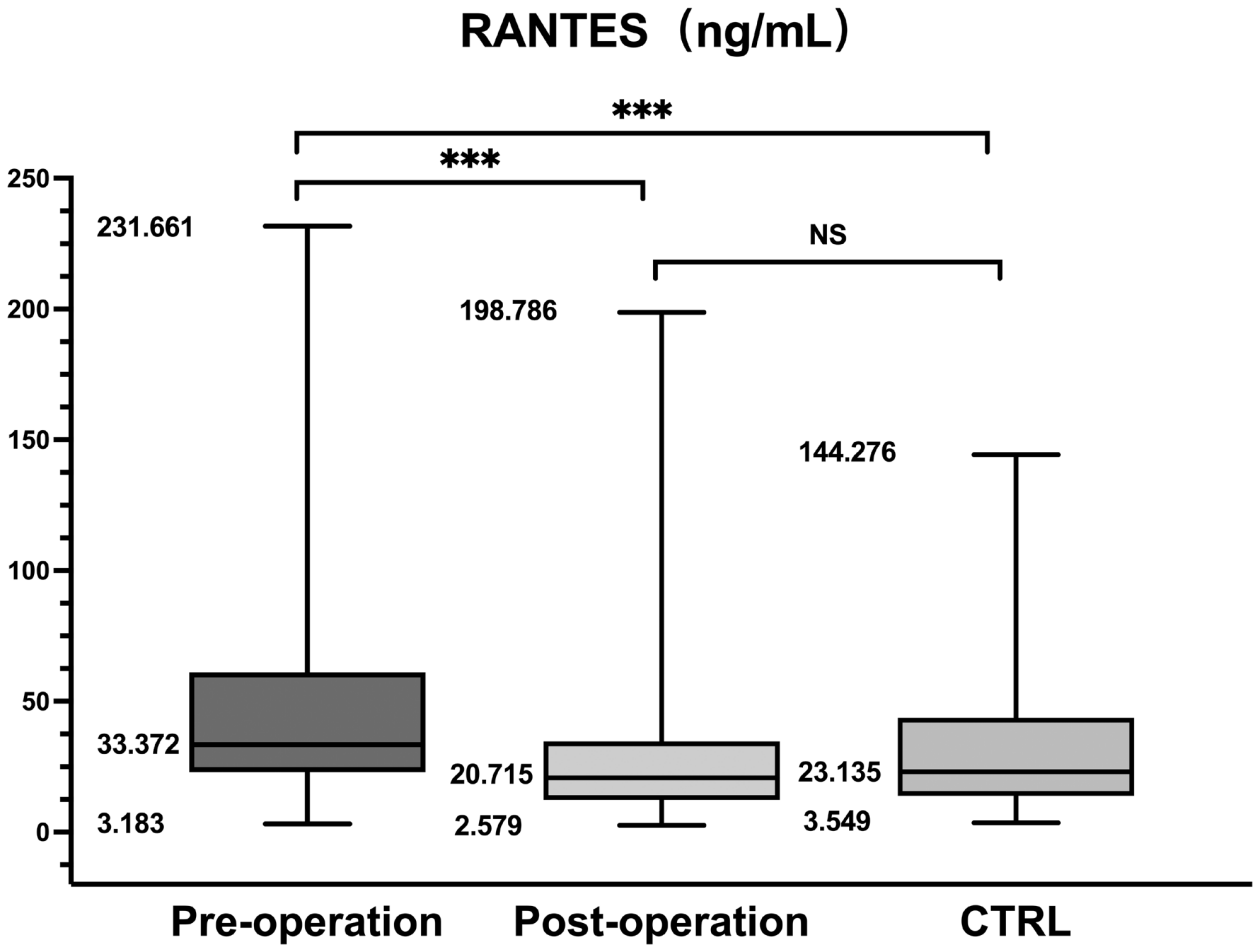
Box and whiskers plot (median, 25–75th percentile, rang) of serum RANTES levels in the PD subgroups (pre‐operation and post‐operation) and CTRL group. The increase in RANTES levels comes mainly from pre‐operative PD patients, while post‐operative patients do not differ significantly compared with the CTRL group.

**FIGURE 4:**
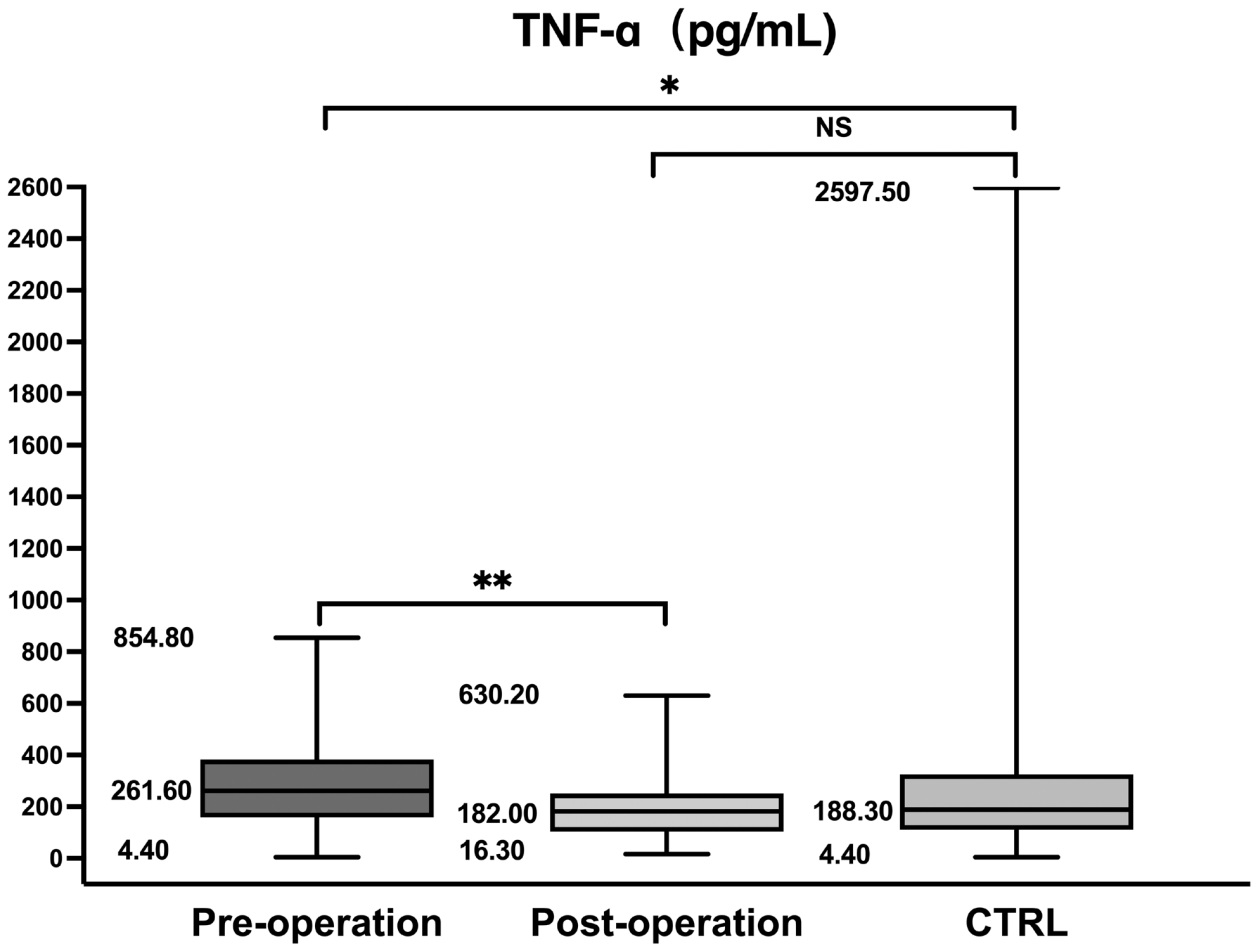
Box and whiskers plot (median, 25–75th percentile, rang) of serum TNF‐α levels in the PD subgroups (pre‐operation and post‐operation) and CTRL group. The result was similar to RANTES levels.

**FIGURE 5:**
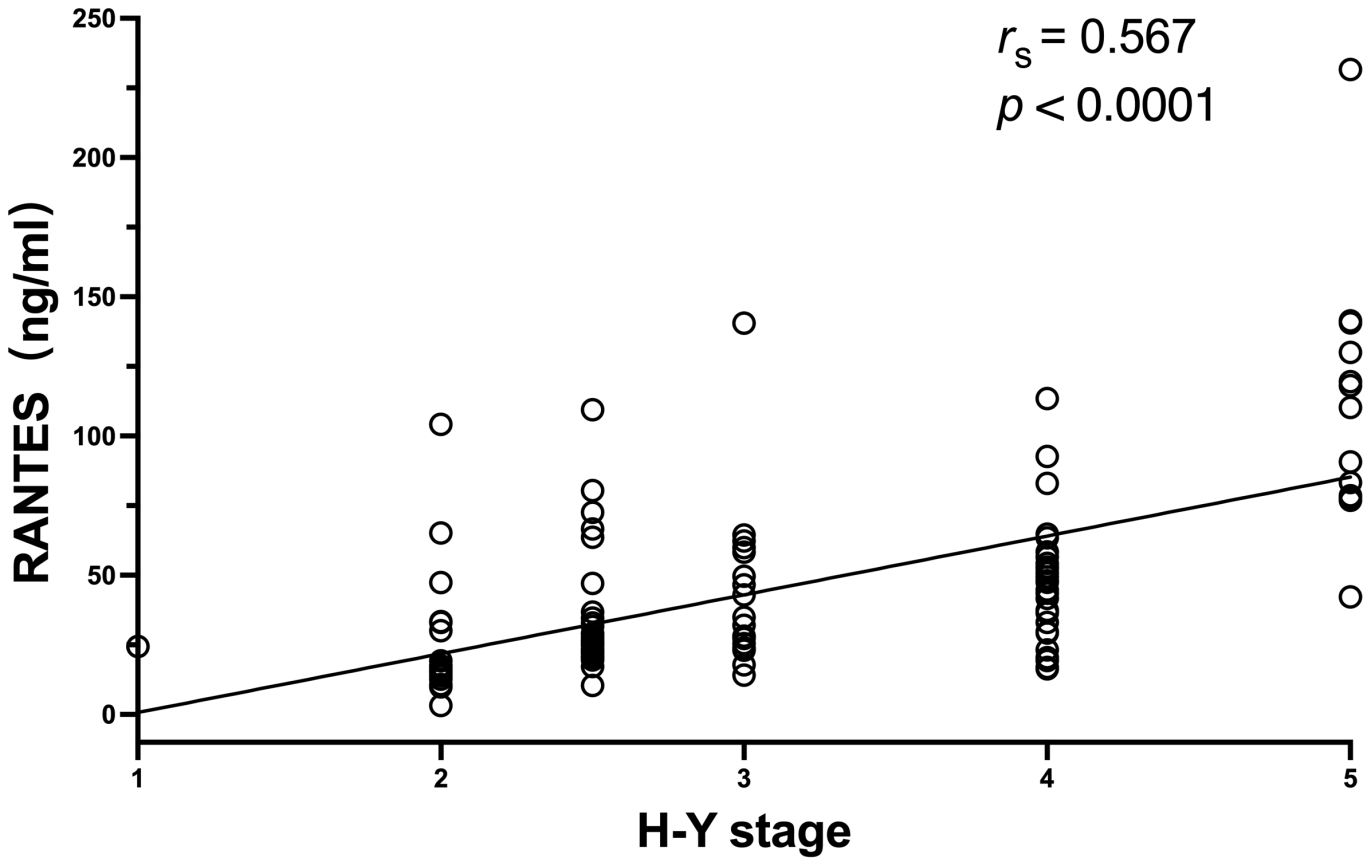
Significant positive correlation between RANTES levels and H‐Y stage in the pre‐operation group (*n* = 113, *r*_s_ = 0.567, *p* < 0.001). *r*_s_, Spearman correlation coefficient.

**FIGURE 6:**
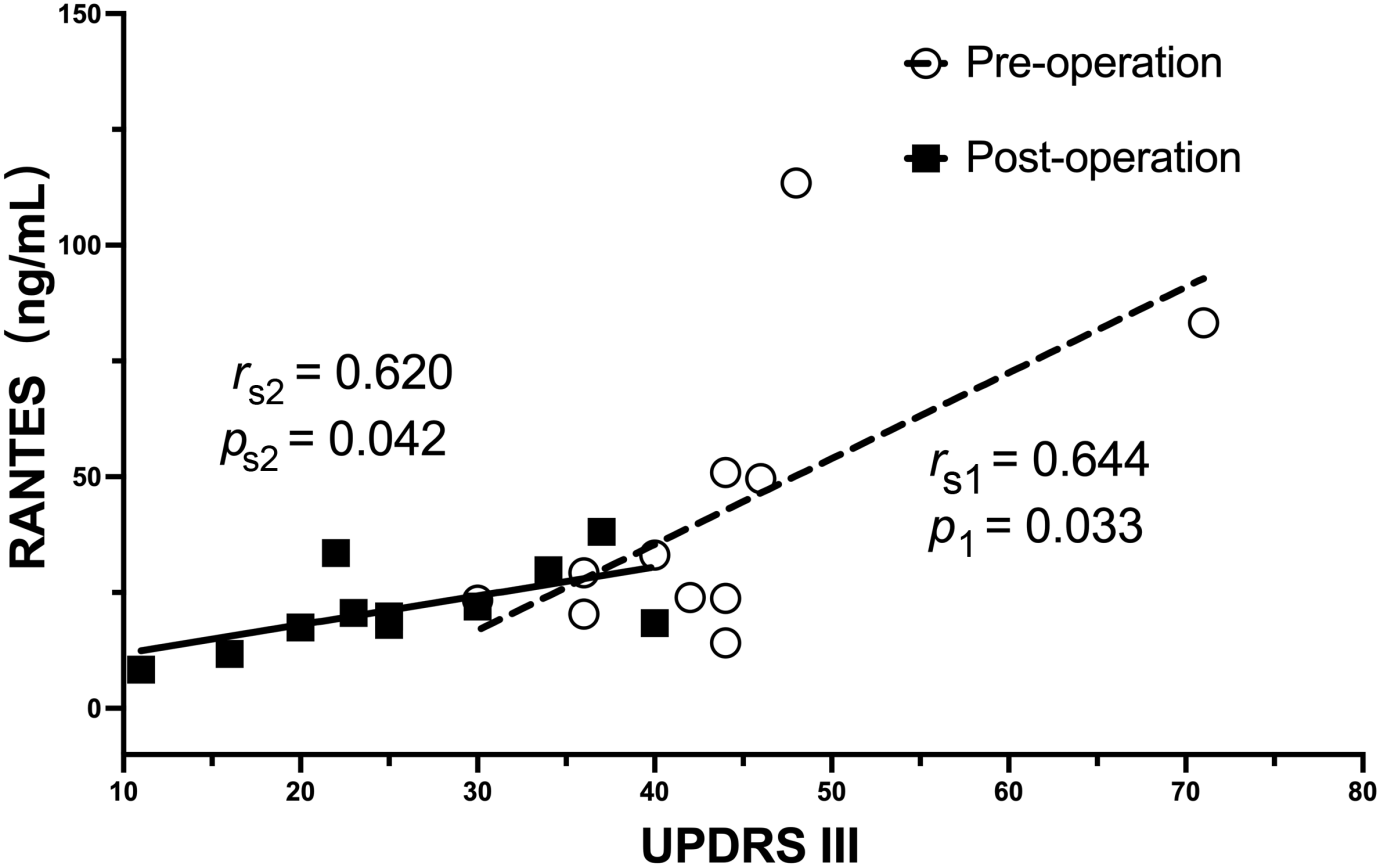
Significant positive correlation between RANTES levels and UPDRS III score in matched PD patients (*n* = 11, *r*_s1_ = 0.644, *p* = 0.033 and *r*_s2_ = 0.620, *p* = 0.042).

The participants' clinical and demographical characteristics are summarized in Table 2. There were no significant differences with respect to sex, age, or H‐Y stage between groups, but there was a significant difference for duration (*p* = 0.005). The PD group showed significantly different RANTES and TNF‐α levels compared with the control group (*p* < 0.001 and *p* = 0.005, respectively). Pairwise comparisons showed significant increases in RANTES and TNF‐α levels between the pre‐operation and post‐operation group and the control group (*p < 0.001, ^△^ *p* < 0.001, ^✦^ *p* = 0.005, ^▲^ *p* = 0.002, Figures 3 and 4). The relationship between H‐Y stage and inflammatory markers levels was only analyzed in the pre‐operation group because there was no change in the other group. Serum RANTES levels were correlated with H‐Y stage in the pre‐operation group, showing a strong positive linear relationship (*n* = 113, *r*_s_ = 0.567, *p* < 0.001, Figure 5); however, TNF‐α levels were not correlated with H‐Y stage. To further explore the interaction, we examined another 11 patients with pre‐operative and post‐operative blood samples (Table 3). The pre‐operation group showed significantly higher UPDRS III score, RANTES, and TNF‐α levels compared to the post‐operation group (*p* = 0.001, *p* = 0.033, and *p* = 0.027). As expected, there were strong and significant correlations between RANTES levels and UPDRS III scores in both groups (*n* = 11, *r*_s1_ = 0.644, *p* = 0.033 and *r*_s2_ = 0.620, *p* = 0.042, Figure 6). TNF‐α and RANTES levels also showed a correlation, although not statistically significant, suggesting a consistent inflammatory response (*n* = 11, *r*_s1_ = 0.545, *p* = 0.083 and *r*_s2_ = 0.636, *p* = 0.035).

**TABLE 2:** Clinical and biochemical data of subjects.

Subjects	Pre‐operation	Post‐operation	CTRL	*p*‐Value
N	109	49	113	
M/F	61/48	24/25	68/45	NS
Age (years)	63.00 (57.50–60.00)	66.00 (56.50–69.00)	63.00 (56.00–68.50)	NS
H‐Y stage	3.00 (2.50–4.00)	3.00 (2.50–4.00)		NS
Duration (years)	7.00 (5.00–12.00)	10.00 (7.00–13.00)		0.005
LED (mg)	600.00 (550.00–697.50)	635.00 (566.70–734.20)		NS
RANTES (ng/mL)	33.37 (22.93–61.16)^*/△^	20.72 (12.25–34.65)	23.14 (13.82–43.74)	<0.001
TNF‐α (pg/mL)	261.60 (158.55–382.70)^✦/▲^	182.00 (103.15–251.20)	188.30 (110.35–325.35)	0.005

*Note*: Data are presented as mean ± SD or median (25th–75th percentile).

Abbreviations: CTRL, control group; H‐Y stage, Hoehn‐Yahr stage; LED, Levodopa Equivalent Dose; *p*‐value, Kruskal–Wallis test; RANTES, regulated on activation, normal T cell expressed and secreted; TNF‐α, tumor necrosis factor‐alpha.

*p < 0.001 and ✦*p* = 0.002 versus post‐operation (Mann–Whitney test); △*p* < 0.001, ▲*p* = 0.020 versus CTRL (Mann–Whitney test); NS between post‐operation and CTRL.

**TABLE 3:** Clinical and biochemical data of matched PD patients.

Subjects	Pre‐operation	Post‐operation	*p*‐Value
*N*	11		
M/F	5/6		
Age (years)	60.55 ± 6.12		
H‐Y stage	3.68 ± 0.72		
Duration (years)	9.82 ± 4.60		
LED (mg)	625.63 ± 116.05	615.03 ± 114.84	NS
UPDRS III	44.00 (36.00–46.00)**	25.00 (20.00–34.00)	0.001
RANTES (ng/mL)	29.27 (23.38–50.92)*	19.87 (17.43–29.83)	0.033
TNF‐α (pg/mL)	249.51 ± 130.78*	134.37 ± 92.43	0.027

*Note*: Data are presented as mean ± SD or median (25th–75th percentile).

Abbreviations: H‐Y stage, Hoehn‐Yahr stage; LED, Levodopa Equivalent Dose; *p*‐value, Mann–Whitney test; RANTES, regulated on activation, normal T cell expressed and secreted; TNF‐α, tumor necrosis factor‐alpha; UPDRS, Unified Parkinson's Disease Rating Scale.

**p = 0.001, *p = 0.033, *p = 0.027 versus post‐operation.

## 4 DISCUSSION

The onset of PD is usually unilateral, presenting with N‐type development. It means static tremor may first appear on one upper or lower extremity, further involving the ipsilateral upper and lower extremities then progressing to the contralateral extremity. However, due to the heterogeneity of PD, the clinical symptoms and treatment methods vary across patients. Accordingly, the diagnosis and treatment of PD requires experienced specialists. New patients are usually treated with medication and achieve some improvement in limb symptoms. However, with progression of the disease, the required drug dosage gradually increases, coupled with the side effects of anti‐Parkinson's drugs, and patients' motor symptoms gradually worsen. Patients may experience symptom fluctuations and dyskinesia, as manifested by sudden onset and cessation of drug effects or involuntary whole‐body writhing at peak blood levels, which exacerbates their pain. In such cases, invasive neuromodulation therapy, that is, DBS surgery, may be recommended. DBS can effectively improve motor fluctuation symptoms, dyskinesia, and slow down progression of the disease, but it does not reflect a complete cure.^29^, ^30^, ^31^ Therefore, the treatment philosophy for PD is to diagnose and treat the disease as early as possible to delay its progression. Due to challenges in the early diagnosis of PD, researchers have aimed to find diagnostic biomarkers.^15^, ^32^, ^33^ In this study of plasma biomarkers in PD patients, we found that RANTES and TNF‐α in blood have potential research value and thus warrant further investigation. Although the results of the screening were not significant, the reasons for this may be the small sample sizes or the possible occult progression of some diseases in the subjects. Our findings also suggest a correlation between plasma biomarkers and surgical efficacy from the perspective of DBS treatment. We will further study the above biomarkers to explore whether the pathogenesis of PD is related to immune inflammatory response and explore the correlation between plasma biomarkers and surgical efficacy, so as to provide clinical basis for further study on the therapeutic mechanism of DBS.

RANTES, also known as C‐C Motif Chemokine Ligand 5 (CCL5), is involved in the human inflammatory immune process. When inflammatory cells secrete RANTES, they tend to involve more T cells in the inflammatory response. Several studies have demonstrated an interaction between systemic inflammation and neuronal damage in neurodegenerative diseases.^34^, ^35^ For example, in the primary neurodegenerative diseases PD and Alzheimer's disease (AD), the initial onset of the disease may be a non‐immune‐mediated injury within the CNS.^36^ Using a PD mouse model, Dutta et al.^37^ demonstrated that RANTES‐induced invasion of Th17 cells into the substantia nigra exacerbated the loss of dopaminergic cells. In the present study, we found that plasma RANTES levels in PD patients were higher than those in healthy subjects. This finding is consistent with previous reports^27^, ^38^ and supports our view that the inflammatory state plays a role in the progression of PD. However, findings on the correlation between plasma RANTES level and disease severity have been inconsistent. Our findings are consistent with those of Tang et al.,^27^ as we found a positive correlation between plasma RANTES level and PD severity (based on H‐Y stage and UPDRS III score) in 11 paired patients. This may be due to increased production of RANTES in the blood of PD patients. Reale et al.^39^ reported that the RANTES content in the supernatant of peripheral blood monocyte culture of PD patients was 1.7 times higher than that of healthy controls, further supporting this theory. Interestingly, after surgical intervention, plasma RANTES levels in PD patients decreased compared to before surgery, and there was no significant difference between post‐surgery patients and healthy controls, suggesting that STN‐DBS (subthalamic nucleus deep brain stimulation) may improve plasma RANTES levels in PD patients. In further analyses, we performed a more refined patient segmentation. We found that the RANTES content in PD patients with rigidity, H‐Y stage 2.5 and 3 patients before and after surgery were not statistically significant but meaningful. The reason that statistical significance was not achieved may be because the sample size was somewhat small.

TNF‐α is an important inflammatory factor that is produced by abnormally activated glial cells during CNS inflammation. Neuroinflammatory events mediated by TNF‐α lead to progressive degeneration of dopaminergic neurons, which plays an important role in the pathogenesis of PD. Previous studies have shown that the concentration of TNF‐α in the brain tissue and cerebrospinal fluid of PD patients is significantly higher than that of controls, suggesting that an immune response may occur in the substantia nigra striatum region of PD patients that is at least partially related to neuronal degeneration.^7^, ^40^, ^41^ Similar results have been reported in animal experiments. For example, long‐term treatment of mice with 1‐Methyl‐4‐phenyl‐1, 2, 3, 6‐tetrahydropyridine/probenecid (MPTPp) resulted in downregulation of Mannose Receptor C‐Type 1 (MRC1) and transforming growth factor‐beta (TGF‐β) expression after cluster of differentiation 11 (CD11) activation, as well as upregulation of TNF‐α and interleukin‐1beta (IL‐1β) expression, suggesting the development of an immune response leading to the death of dopaminergic neurons.^42^ In contrast, knockdown of the TNF‐α receptor gene had a protective effect in PD mice.^42^ In the present study, we found a significant difference in TNF‐α levels between PD patients and healthy subjects, and this difference disappeared between patients and healthy subjects after surgery. Furthermore, we observed a correlation between TNF‐α and RANTES levels, suggesting a synergistic effect of these two plasma proteins in PD. TNF‐α may induce the expression of chemokines such as C‐X‐C Motif Chemokine Ligand 6 (CXCL6). However, there are no studies that address the correlation between chemokines and PD. In our study, there was a lack of association between TNF‐α levels and disease severity. We cannot exclude the effects of chance or individual variation in our study. Increasing the sample size or measuring the clearance rate of TNF‐α may help to explain this unexpected result.

By comparing changes in plasma RANTES and TNF‐α levels in PD patients before and after surgery for the first time, our findings suggest that DBS may delay the progression of PD by improving the systemic immune inflammatory response. In studies of levodopa, levodopa or dopamine agonists appeared to prevent the development of immune disorders, as serum RANTES levels were significantly higher in untreated subjects compared to subjects or treated PD patients. We speculate that DBS may have similar effects, such as reducing inflammatory factors in the body by improving the systemic immune inflammatory response of PD patients, thus alleviating further damage to neurons. However, further research on the cellular and molecular mechanisms is warranted. In fact, other related proinflammatory factors have been compared, but the results are not presented here. For example, for proteins such as IL‐6 and CRP, our experimental results did not show significant differences between before and after intervention. In some experiments, though, the researchers detected differences in these proteins.^27^ However, IL‐6 and CRP concentrations that were found to be increased in PD have also been shown to be in normal physiology and increased in the aged brain.^43^ Evidence has been presented to show that chronic neuroinflammation occurs in the aging brain which are hallmarked by elevated immunoreactivity and low‐scale constant production of cytokines.^44^ We believe that this concomitant effect must be considered. In the subsequent research, we will focus on exploring the relationship between them.

## 5 LIMITATIONS

The study still has some limitations. As this study was a retrospective study and the surgical methods and procedures were optimized, more samples could not be added to support the experimental results. In addition, due to the inadequate study design, which resulted in a small number of matched patients, further studies should have more matched patients. Finally, the relationship between inflammatory proteins associated with the nervous system still needs to be supported by more experimental results. Systematic evaluation should be included in future studies.

## 6 CONCLUSIONS

This study showed significant differences in serum RANTES and TNF‐α levels in PD patients compared to matched controls. Our findings indicate that PD patients have an on‐going systemic inflammatory profile and that RANTES and TNF‐α levels are potential plasma biomarkers for PD. Moreover, elevated RANTES and TNF‐α levels were associated with the severity of PD and, in paired comparisons, RANTES levels correlated with UPDRS scores. These results support the effectiveness of DBS for PD and suggest that its mechanism of action may involve altering the systemic inflammatory response. Unfortunately, we were unable to further explore cellular‐molecular mechanisms. The source of plasma inflammatory factors in PD patients should be elucidated in future studies and the mechanism of DBS should be further investigated to contribute to delaying or even curing PD.

## AUTHOR CONTRIBUTIONS

All authors contributed to the study conception and design. Data collection and analysis were performed by BL, CQ, and YL. The first draft of the manuscript was written by LC and WWD. WBZ edited and revised the manuscript. All authors contributed to and approved the final manuscript.

## FUNDING INFORMATION

This study was supported by the grant from Subtopic of the 13th Five‐Year National Key Research and Development Plan (No. 2016YFC0105901NNZ), Special Funds of the Jiangsu Provincial Key Research and Development Projects (No. BE2022049, BE2022049‐1), and Nanjing Health Science and Technology Development Special Fund Project (No. ZKX20031).

## CONFLICT OF INTEREST STATEMENT

The authors declare that they have no competing interests.

## CONSENT TO PARTICIPATE

All patients or their family members provided written informed consent for participation in the study.

## CONSENT FOR PUBLICATION

Not applicable.

## Supporting information


Appendix S1

## Data Availability

The original contributions presented in the study are included in the article/Supplementary Material, further inquiries can be directed to the corresponding authors.

## References

[1] Bloem BR , Okun MS , Klein C . Parkinson's disease. Lancet. 2021;397(10291):2284‐2303. 33848468 10.1016/S0140-6736(21)00218-X

[2] Balestrino R , Schapira AHV . Parkinson disease. Eur J Neurol. 2020;27(1):27‐42. 31631455 10.1111/ene.14108

[3] Polymeropoulos MH , Lavedan C , Leroy E , et al. Mutation in the alpha‐synuclein gene identified in families with Parkinson's disease. Science. 1997;276(5321):2045‐2047. 9197268 10.1126/science.276.5321.2045

[4] de Meira Santos Lima M , Braga Reksidler A , Marques Zanata S , Bueno Machado H , Tufik S , Vital MABF . Different parkinsonism models produce a time‐dependent induction of COX‐2 in the substantia nigra of rats. Brain Res. 2006;1101(1):117‐125. 16781689 10.1016/j.brainres.2006.05.016

[5] Mollenhauer B , Trautmann E , Taylor P , et al. Total CSF α‐synuclein is lower in de novo Parkinson patients than in healthy subjects. Neurosci Lett. 2013;532:44‐48. 23149132 10.1016/j.neulet.2012.11.004

[6] Kang J‐H , Irwin DJ , Chen‐Plotkin AS , et al. Association of cerebrospinal fluid β‐amyloid 1‐42, T‐tau, P‐tau181, and α‐synuclein levels with clinical features of drug‐naive patients with early Parkinson disease. JAMA Neurol. 2013;70(10):1277‐1287. 23979011 10.1001/jamaneurol.2013.3861 PMC4034348

[7] Whitton PS . Inflammation as a causative factor in the aetiology of Parkinson's disease. Br J Pharmacol. 2007;150(8):963‐976. 17339843 10.1038/sj.bjp.0707167 PMC2013918

[8] Aktas O , Ullrich O , Infante‐Duarte C , Nitsch R , Zipp F . Neuronal damage in brain inflammation. Arch Neurol. 2007;64(2):185‐189. 17296833 10.1001/archneur.64.2.185

[9] Kannarkat GT , Boss JM , Tansey MG . The role of innate and adaptive immunity in Parkinson's disease. J Parkinsons Dis. 2013;3(4):493‐514. 24275605 10.3233/JPD-130250 PMC4102262

[10] Harms AS , Ferreira SA , Romero‐Ramos M . Periphery and brain, innate and adaptive immunity in Parkinson's disease. Acta Neuropathol. 2021;141(4):527‐545. 33555429 10.1007/s00401-021-02268-5 PMC7952334

[11] Sabatino JJ , Pröbstel A‐K , Zamvil SS . B cells in autoimmune and neurodegenerative central nervous system diseases. Nat Rev Neurosci. 2019;20(12):728‐745. 31712781 10.1038/s41583-019-0233-2

[12] Shulman JM , De Jager PL , Feany MB . Parkinson's disease: genetics and pathogenesis. Annu Rev Pathol. 2011;6:193‐222. 21034221 10.1146/annurev-pathol-011110-130242

[13] El‐Agnaf OMA , Salem SA , Paleologou KE , et al. Detection of oligomeric forms of alpha‐synuclein protein in human plasma as a potential biomarker for Parkinson's disease. FASEB J. 2006;20(3):419‐425. 16507759 10.1096/fj.03-1449com

[14] Emmer KL , Waxman EA , Covy JP , Giasson BI . E46K human alpha‐synuclein transgenic mice develop Lewy‐like and tau pathology associated with age‐dependent, detrimental motor impairment. J Biol Chem. 2011;286(40):35104‐35118. 21846727 10.1074/jbc.M111.247965 PMC3186371

[15] Abdi IY , Ghanem SS , El‐Agnaf OM . Immune‐related biomarkers for Parkinson's disease. Neurobiol Dis. 2022;170:105771. 35598675 10.1016/j.nbd.2022.105771

[16] Hu X , Leak RK , Shi Y , et al. Microglial and macrophage polarization—new prospects for brain repair. Nat Rev Neurol. 2015;11(1):56‐64. 25385337 10.1038/nrneurol.2014.207 PMC4395497

[17] Zhang Q‐S , Heng Y , Yuan Y‐H , Chen N‐H . Pathological α‐synuclein exacerbates the progression of Parkinson's disease through microglial activation. Toxicol Lett. 2017;265:30‐37. 27865851 10.1016/j.toxlet.2016.11.002

[18] De Virgilio A , Greco A , Fabbrini G , et al. Parkinson's disease: autoimmunity and neuroinflammation. Autoimmun Rev. 2016;15(10):1005‐1011. 27497913 10.1016/j.autrev.2016.07.022

[19] Brochard V , Combadière B , Prigent A , et al. Infiltration of CD4+ lymphocytes into the brain contributes to neurodegeneration in a mouse model of Parkinson disease. J Clin Invest. 2009;119(1):182‐192. 19104149 10.1172/JCI36470 PMC2613467

[20] Appay V , Rowland‐Jones SL . RANTES: a versatile and controversial chemokine. Trends Immunol. 2001;22(2):83‐87. 11286708 10.1016/s1471-4906(00)01812-3

[21] Luster AD . Chemokines–chemotactic cytokines that mediate inflammation. N Engl J Med. 1998;338(7):436‐445. 9459648 10.1056/NEJM199802123380706

[22] Moser B , Loetscher P . Lymphocyte traffic control by chemokines. Nat Immunol. 2001;2(2):123‐128. 11175804 10.1038/84219

[23] Charo IF , Ransohoff RM . The many roles of chemokines and chemokine receptors in inflammation. N Engl J Med. 2006;354(6):610‐621. 16467548 10.1056/NEJMra052723

[24] Zhong J , Fan S , Yan Z , et al. Effects of Nogo‐A silencing on TNF‐α and IL‐6 secretion and TH downregulation in lipopolysaccharide‐stimulated PC12 cells. Biomed Res Int. 2015;2015:817914. 26583134 10.1155/2015/817914 PMC4637059

[25] Rai SN , Birla H , Zahra W , Singh SS , Singh SP . Immunomodulation of Parkinson's disease using Mucuna pruriens (Mp). J Chem Neuroanat. 2017;85:27‐35. 28642128 10.1016/j.jchemneu.2017.06.005

[26] Kouchaki E , Kakhaki RD , Tamtaji OR , et al. Increased serum levels of TNF‐α and decreased serum levels of IL‐27 in patients with Parkinson disease and their correlation with disease severity. Clin Neurol Neurosurg. 2018;166:76‐79. 29408778 10.1016/j.clineuro.2018.01.022

[27] Tang P , Chong L , Li X , et al. Correlation between serum RANTES levels and the severity of Parkinson's disease. Oxid Med Cell Longev. 2014;2014:208408. 25587378 10.1155/2014/208408 PMC4283268

[28] Brodacki B , Staszewski J , Toczyłowska B , et al. Serum interleukin (IL‐2, IL‐10, IL‐6, IL‐4), TNFalpha, and INFgamma concentrations are elevated in patients with atypical and idiopathic parkinsonism. Neurosci Lett. 2008;441(2):158‐162. 18582534 10.1016/j.neulet.2008.06.040

[29] Castrioto A , Lozano AM , Poon Y‐Y , Lang AE , Fallis M , Moro E . Ten‐year outcome of subthalamic stimulation in Parkinson disease: a blinded evaluation. Arch Neurol. 2011;68(12):1550‐1556. 21825213 10.1001/archneurol.2011.182

[30] St George RJ , Carlson‐Kuhta P , Burchiel KJ , Hogarth P , Frank N , Horak FB . The effects of subthalamic and pallidal deep brain stimulation on postural responses in patients with Parkinson disease. J Neurosurg. 2012;116(6):1347‐1356. 22424564 10.3171/2012.2.JNS11847 PMC3465575

[31] Rizzone MG , Fasano A , Daniele A , et al. Long‐term outcome of subthalamic nucleus DBS in Parkinson's disease: from the advanced phase towards the late stage of the disease? Parkinsonism Relat Disord. 2014;20(4):376‐381. 24508574 10.1016/j.parkreldis.2014.01.012

[32] Fang F , Zhan Y , Hammar N , et al. Lipids, apolipoproteins, and the risk of Parkinson disease. Circ Res. 2019;125(6):643‐652. 31382822 10.1161/CIRCRESAHA.119.314929

[33] Rahmani F , Saghazadeh A , Rahmani M , et al. Plasma levels of brain‐derived neurotrophic factor in patients with Parkinson disease: a systematic review and meta‐analysis. Brain Res. 2019;1704:127‐136. 30296429 10.1016/j.brainres.2018.10.006

[34] Holmans P , Moskvina V , Jones L , et al. A pathway‐based analysis provides additional support for an immune‐related genetic susceptibility to Parkinson's disease. Hum Mol Genet. 2013;22(5):1039‐1049. 23223016 10.1093/hmg/dds492 PMC3561909

[35] Collins LM , Toulouse A , Connor TJ , Nolan YM . Contributions of central and systemic inflammation to the pathophysiology of Parkinson's disease. Neuropharmacology. 2012;62(7):2154‐2168. 22361232 10.1016/j.neuropharm.2012.01.028

[36] Voet S , Srinivasan S , Lamkanfi M , van Loo G . Inflammasomes in neuroinflammatory and neurodegenerative diseases. EMBO Mol Med. 2019;11(6):e10248. 31015277 10.15252/emmm.201810248 PMC6554670

[37] Dutta D , Kundu M , Mondal S , et al. RANTES‐induced invasion of Th17 cells into substantia nigra potentiates dopaminergic cell loss in MPTP mouse model of Parkinson's disease. Neurobiol Dis. 2019;132:104575. 31445159 10.1016/j.nbd.2019.104575 PMC6834904

[38] Rentzos M , Nikolaou C , Andreadou E , et al. Circulating interleukin‐15 and RANTES chemokine in Parkinson's disease. Acta Neurol Scand. 2007;116(6):374‐379. 17986095 10.1111/j.1600-0404.2007.00894.x

[39] Reale M , Iarlori C , Thomas A , et al. Peripheral cytokines profile in Parkinson's disease. Brain Behav Immun. 2009;23(1):55‐63. 18678243 10.1016/j.bbi.2008.07.003

[40] McGeer PL , Itagaki S , Boyes BE , McGeer EG . Reactive microglia are positive for HLA‐DR in the substantia nigra of Parkinson's and Alzheimer's disease brains. Neurology. 1988;38(8):1285‐1291. 3399080 10.1212/wnl.38.8.1285

[41] Mogi M , Harada M , Riederer P , Narabayashi H , Fujita K , Nagatsu T . Tumor necrosis factor‐alpha (TNF‐alpha) increases both in the brain and in the cerebrospinal fluid from parkinsonian patients. Neurosci Lett. 1994;165(1–2):208‐210. 8015728 10.1016/0304-3940(94)90746-3

[42] Lecca D , Janda E , Mulas G , et al. Boosting phagocytosis and anti‐inflammatory phenotype in microglia mediates neuroprotection by PPARγ agonist MDG548 in Parkinson's disease models. Br J Pharmacol. 2018;175(16):3298‐3314. 29570770 10.1111/bph.14214 PMC6057897

[43] Puzianowska‐Kuźnicka M , Owczarz M , Wieczorowska‐Tobis K , et al. Interleukin‐6 and C‐reactive protein, successful aging, and mortality: the PolSenior study. Immun Ageing. 2016;13:21. 27274758 10.1186/s12979-016-0076-x PMC4891873

[44] Hsu B , Hirani V , Cumming RG , et al. Cross‐sectional and longitudinal relationships between inflammatory biomarkers and frailty in community‐dwelling older men: the Concord Health and Ageing in Men Project. J Gerontol A Biol Sci Med Sci. 2019;74(6):835‐841. 28977375 10.1093/gerona/glx142

